# Supplementary material for: The interactome of intact mitochondria by cross-linking mass spectrometry provides evidence for coexisting respiratory supercomplexes
Source: Mol Cell Proteomics. 2017 Dec 8;17(2):216–32. doi: 10.1074/mcp.RA117.000470 (PMC5795388; doi:10.1074/mcp.RA117.000470)
Supplement: Supplemental Data [file supp_RA117.000470_133922_0_supp_23978_fzffwf.zip › spectra_annotation/mito_spectra_annotation/188-1-1-1-5-1.pdf]

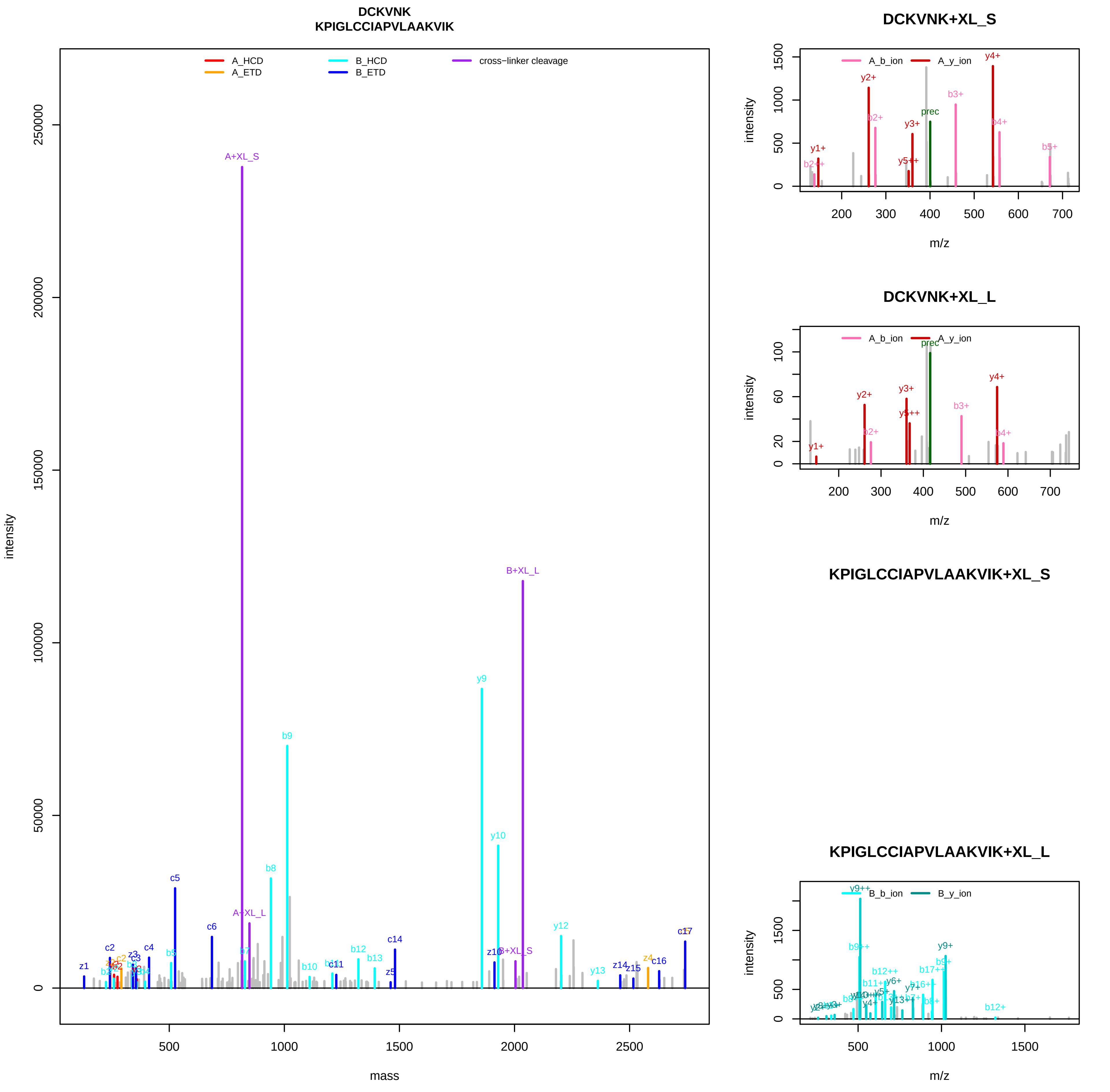

DCKVNK
KPIGLCCIAPVLAAKVIK
A_HCD
A_ETD
B_HCD
B_ETD
cross−linker cleavage
A+XL_S
A+XL_L
B+XL_L
B+XL_S
y9
y10
y12
y13
b5
b7
b8
b9
b10
b11
b12
b13
c2
c3
c4
c5
c6
c11
c14
c16
c17
z1
z3
z4
z5
z10
z14
z15
intensity
mass
DCKVNK+XL_S
A_b_ion
A_y_ion
y1+
y2+
y3+
y4+
y5++
b2+
b2++
b3+
b4+
b5+
prec
m/z
DCKVNK+XL_L
KPIGLCCIAPVLAAKVIK+XL_S
KPIGLCCIAPVLAAKVIK+XL_L
B_b_ion
B_y_ion
y9++
y9+
b9++
b9+
b12++
b12+
b11++
b16++
b17++
y6+
y5+
y4+
y7+
y13++
y10++
b8+
